# Considerations for Polymers Used in Ocular Drug Delivery

**DOI:** 10.3389/fmed.2021.787644

**Published:** 2022-01-28

**Authors:** Megan M. Allyn, Richard H. Luo, Elle B. Hellwarth, Katelyn E. Swindle-Reilly

**Affiliations:** ^1^William G. Lowrie Department of Chemical and Biomolecular Engineering, The Ohio State University, Columbus, OH, United States; ^2^Department of Biomedical Engineering, The Ohio State University, Columbus, OH, United States; ^3^Department of Ophthalmology and Visual Sciences, The Ohio State University, Columbus, OH, United States

**Keywords:** drug delivery, polymer, hydrogel, ophthalmic delivery, ocular implants, controlled release, ocular biomaterials

## Abstract

**Purpose:**

Age-related eye diseases are becoming more prevalent. A notable increase has been seen in the most common causes including glaucoma, age-related macular degeneration (AMD), and cataract. Current clinical treatments vary from tissue replacement with polymers to topical eye drops and intravitreal injections. Research and development efforts have increased using polymers for sustained release to the eye to overcome treatment challenges, showing promise in improving drug release and delivery, patient experience, and treatment compliance. Polymers provide unique properties that allow for specific engineered devices to provide improved treatment options. Recent work has shown the utilization of synthetic and biopolymer derived biomaterials in various forms, with this review containing a focus on polymers Food and Drug Administration (FDA) approved for ocular use.

**Methods:**

This provides an overview of some prevalent synthetic polymers and biopolymers used in ocular delivery and their benefits, brief discussion of the various types and synthesis methods used, and administration techniques. Polymers approved by the FDA for different applications in the eye are listed and compared to new polymers being explored in the literature. This article summarizes research findings using polymers for ocular drug delivery from various stages: laboratory, preclinical studies, clinical trials, and currently approved. This review also focuses on some of the challenges to bringing these new innovations to the clinic, including limited selection of approved polymers.

**Results:**

Polymers help improve drug delivery by increasing solubility, controlling pharmacokinetics, and extending release. Several polymer classes including synthetic, biopolymer, and combinations were discussed along with the benefits and challenges of each class. The ways both polymer synthesis and processing techniques can influence drug release in the eye were discussed.

**Conclusion:**

The use of biomaterials, specifically polymers, is a well-studied field for drug delivery, and polymers have been used as implants in the eye for over 75 years. Promising new ocular drug delivery systems are emerging using polymers an innovative option for treating ocular diseases because of their tunable properties. This review touches on important considerations and challenges of using polymers for sustained ocular drug delivery with the goal translating research to the clinic.

## Introduction

In 2020, the World Health Organization reported 196 million cases of age-related macular degeneration (AMD), 146 million cases of diabetic retinopathy (DR), 76 million cases of glaucoma, and 65 million cases of cataract globally (1). Nearly 44 million people in the United States (US) over the age of 40 are afflicted by some form of eye disease, with 2010 National Eye Institute statistics showing 24 million cataract cases, 11 million early and late stage AMD cases, 7 million DR cases, and 2 million glaucoma cases (2–4). While each disease has unique causes, symptoms, and treatments, all will result in complete vision loss if left untreated. Difficulties in ocular drug delivery stem from the complex anatomy of the eye, its compartmentalization, and its separation from the rest of the body.

Ocular anatomy is generally classified into two segments: the anterior segment comprised of the iris, cornea, lens, and surrounding aqueous humor; and the posterior segment including the vitreous humor, retina, macula, and optic nerve (5). Cataract and glaucoma impact the lens and fluid drainage pathways, respectively, in the anterior segment. The lens is responsible for accommodation, or fine focusing of light to produce vision. The proteins within the lens work to manage absorbed UV light to maintain the oxidative balance necessary for proper function. With age, the ability of the lens to mitigate oxidative damage and repair cellular damage diminishes, causing protein aggregation, lens opacities, and eventual vision loss due to cataract (6). Though not yet fully understood, it is believed that glaucoma develops when anterior fluid drainage systems based in the trabecular meshwork and uveoscleral outflow pathway become imbalanced (7). Impaired drainage causes an increase in intraocular pressure (IOP) that applies stress on anterior and posterior segments of the eye. Increased posterior IOP can subject the lamina cribrosa to conformational changes that inhibit axonal signal transportation to the optic nerve, resulting in retinal ganglion cell death and vision loss (7). This increased IOP has been theorized to also impact the cornea and corneal endothelium. In patients with angle-closure glaucoma, high IOP was found to cause up to an 11% decrease in endothelial cell density (8). Other common anterior segment disorders affecting the cornea include dry eye disease, corneal neovascularization, anterior uveitis, and keratitis.

Visual disorders affecting the posterior segment include retinal diseases such as AMD, non-proliferative and proliferative diabetic retinopathy (PDR), diabetic macular edema (DME), and posterior conjunctivitis. These present additional difficulties in treatment due to limited accessibility of disease sites and complexity of disease progression. In AMD, disease propagation occurs from an increased inflammatory environment caused by accumulation of reactive oxygen species (ROS) within the retina that leads to protein aggregation and drusen formation. This signals a local over-production of vascular endothelial growth factor (VEGF) that leads to abnormal blood vessel growth, permanently impacting the retinal pigment epithelium (RPE), leading to late stage “wet” AMD (9, 10). Posterior segment diseases resulting from diabetes stem from increased levels of glucose in the bloodstream that cause pericyte apoptosis, outpouching of the capillaries, and the eventual development of microaneurysms. Hyperglycemic oxidative stress and chronic inflammation stimulate the expression of signaling cytokines that increase the permeability of the endothelium to VEGF, resulting in neovascularization, breakdown of the blood-retinal barrier (BRB), and neuronal degradation (11, 12).

Approved therapeutics for treatment of ocular diseases include steroids such as hydrocortisone, triamcinolone acetonide, fluocinolone, and dexamethasone; antibiotics such as fluoroquinolones, tetracyclines, and aminoglycosides; and biological pharmaceuticals such as anti-VEGFs. Experimental therapeutics currently being investigated include antioxidants such as glutathione and ascorbic acid, complement factor inhibitors such as avacincaptad pegol and APL-2, and novel therapeutic mechanisms such as mesenchymal stem cell extracellular vesicles, miotic based eye drops, viral vectors for gene therapy, and adenosine receptors (13–18).

Local delivery is often a necessity in the eye due to the BRB. However, direct administration through eye drops, subconjunctival injection, or intravitreal injection provide only short-term relief and require frequent administration, with outcomes heavily relying on patient compliance (19). Newly emerging ocular drug delivery technology has focused on the use of polymeric biomaterials to address the present obstacles within the field. Several current treatments rely on polymers to extend release duration in the eye, and extensive research is being conducted with current and investigational therapeutics to reduce application frequency.

In this review, a polymeric biomaterial is defined as large macromolecule composed of building blocks being applied in a biomedical application. These building blocks can be composed of synthetic monomers and/or natural components such as amino acids or sugars. These building blocks, in addition to polymer processing techniques, provide tunable chemical and physical characteristics to improve drug delivery and/or dosing of the therapeutic. Polymers are currently being used clinically to increase solubility of a drug in the target environment, control release rates of therapeutics, and improve drug retention within the eye (5, 20). The variety of polymers available to be used in drug delivery are vast but generally fall into two categories: synthetic polymers or biopolymers. The differences, benefits, and specific uses for both types are summarized below.

## Synthetic Polymeric Biomaterials

**Figure 1:**
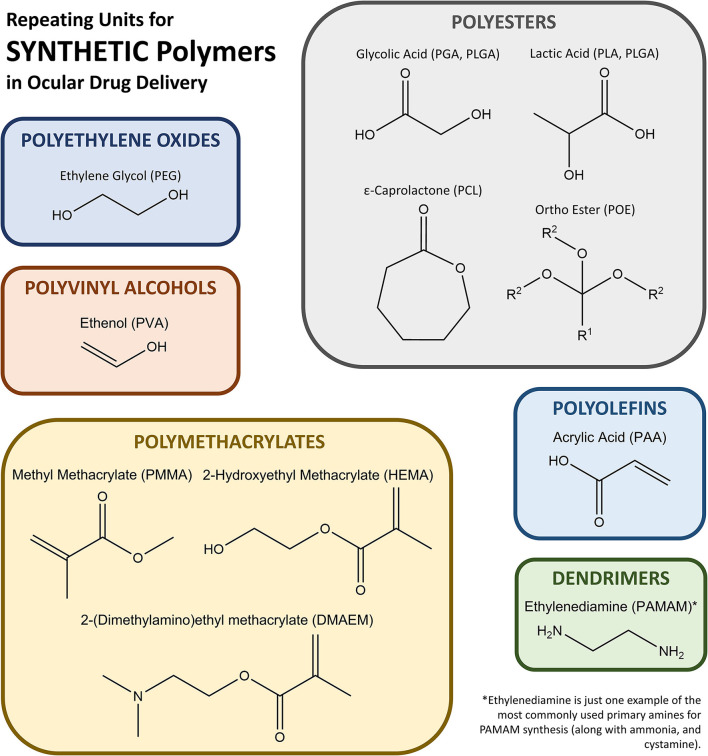
Schematic of monomers that form the most common synthetic polymers used in ocular drug delivery.

Synthetic polymers are based on chemically derived monomers and provide a plethora of mechanical, chemical, and degradation options when utilized for ocular drug delivery applications. Notable synthetic polymers that are US Food and Drug Administration (FDA) approved for ocular applications and in clinical use include poly(ethylene glycol) (PEG), poly(vinyl alcohol) (PVA), poly(glycolic acid) (PGA), poly(lactic-co-glycolic acid) (PLGA), poly[2-(dimethylamino)ethyl methacrylate] (DMAEM), poly(caprolactone) (PCL), poly(acrylic acid) (PAA), and poly(amidoamine) (PAMAM), but many other polymers are available for experimental use or have been approved for use in different applications outside the eye. Monomers used to synthesize most of the synthetic polymers of interest are shown in Figure 1.

### Polyethylene Oxides

PEG is a clear, colorless hydrophilic polymer based on ethylene oxide monomers that can increase the biocompatibility, solubility, and bioavailability of incorporated therapeutics. PEG is available in many forms (liquid, solid), molecular weights, configurations (e.g., linear vs. multi-armed), and activities (e.g., bioinert, tetrafunctional). It is generally regarded as safe (GRAS) by the FDA and is approved for many applications including ophthalmic use. Macugen® is a PEGylated oligoribonucleotide, approved for use in 2004, that possesses a high binding affinity for VEGFs and is an injectable treatment for late stage AMD (21, 22). Shorter term implants such as Dextenza®, a PEG-based cylindrical implant, utilize the controlled release properties of PEG by slowing hydrolytic erosion to provide 1 month of dexamethasone release for both inflammation and pain management after surgery. The device is implanted through intracanalicular insertion, has been FDA approved since 2018 for its indicated use, and is currently in phase 3 clinical trials for treatment of allergic conjunctivits (23). Additional products from Ocular Therapeutix, such as their dexamethasone intracanalicular ophthalmic insert (OTX-DED) and tyrosine kinase inhibitor (OTX-TKI), use PEG. OTX-DED is a smaller dose, shorter duration therapeutic based on the same PEG technology as Dextenza® for delivering dexamethasone (24). The implant is an intracanalicular insert that is currently in phase 2 clinical trials for treatment of dry eye disease (24). OTX-TKI is an *in situ* forming injectable PEG hydrogel for sustained release of axitinib for treatment of wet AMD. The implant was developed to provide therapeutic for up to 12 months and is currently in phase 1 clinical trials (25). Topically applied ReSure® sealant by Ocular Therapeutix is a *in situ* forming PEG hydrogel used to prevent wound leakage after cataract surgery (26). Experimental work done by Foroutan et al. (27) and Hussein et al. (28) confirms that incorporation of PEG units or PEGylation increases the bioavailability and corneal absorption of topically applied steroids by steric hinderance of hydrolytic enzymes and enhanced adherence to the subconjunctiva, reducing drug loss during tear clearance. In intravitreal injections, PEGylation has shown to improve the half-life of hydrophobic therapeutics in the vitreous, allowing for a longer, more effective targeting to the posterior of the eye (29). Further experimental work was completed by Lakhani et al. (30) and showed the incorporation of PEG in nanostructured lipid carriers helped to solubilize amphotericin B, an effective anti-mycotic compound for topical treatment of ocular fungal infections (30). PEG is currently used clinically, in clinical trials, and being investigated for both delivery systems and PEGylation of therapeutics.

### Polyvinyl Alcohols

PVA is a water soluble, biodegradable polymer often used for solubilizing hydrophobic drugs, providing chemical resistivity and ease of processing (31). Variations in synthesis methods allow PVA to be obtained in a range of hydration states and molecular weights (31). PVA's polymer structure provides tunable permeability for controlled release applications. It is on the FDA GRAS list and is currently employed in several nondegradable implants used in the eye, including Vitrasert®, Retisert®, and Iluvien®. Vitrasert® was the first FDA approved PVA and ethylene-vinyl acetate (EVA) copolymer implant for intravitreal treatment of cytomegalovirus retinitis, providing 6–8 months of therapeutic dosing through passive diffusive release from the implant (32). Retisert® is a multi-layered implant that uses a permeable PVA outer layer to provide controlled release of fluocinolone acetonide for up to 2.5 years for treatment of non-infectious posterior uveitis (32, 33). Iluvien® was recently FDA approved for treatment of DME, and the delivery technology is in clinical trials for treatment of additional ocular conditions such as wet AMD. The PVA-based implant provides controlled release of fluocinolone acetonide to the vitreous chamber for up to 36 months (34–36). While similar in composition and eluted therapeutic to Retisert®, implantation of Iluvien® can be completed in out-patient facilities due to its smaller size, reducing surgical risks (37). Yutiq® is another recently approved polyimide/PVA implant for controlled release of fluocinolone acetonide, improving not only on the administration method of both Retisert® and Iluvien®, but also on the ability to treat uveitis (38). PVA is also being evaluated for topical ocular drug delivery through wafers (39).

### Polyesters

PGA, PLA, and PLGA are the most common synthetic polymers in drug delivery vehicles used to treat ocular diseases and are FDA approved for ocular use. These hydrophobic polyesters provide tunable *in vivo* biodegradation based on the monomer ratio incorporated in the polymer, and have seen usage as drug carriers for small molecules, proteins, and genes (40). PLA and PGA are composed of naturally sourced lactic acid and glycolic acid monomers; both are synthesized through ring-opening polymerization (41). Due to its susceptibility to hydrolysis, PGA has a faster degradation rate compared to PLA, which has an *in vivo* biodegradation rate of up to 2 years, but biodegradation of both polymers produces non-toxic byproducts (42). PLA has high thermal stability and can be formed by many methods, i.e., injection molding and extrusion (42). PGA is brittle and insoluble in common organic solvents, thus limiting its processing as a standalone polymer; however, it is often used in conjunction with other polymers. Utilization of PLA's slow biodegradation rate can be seen in Brimo DDS®, an intravitreal implant containing PLA for slow release of brimonidine for treatment of geographic atrophy which has completed phase 2 clinical trials (43).

**Figure 2:**
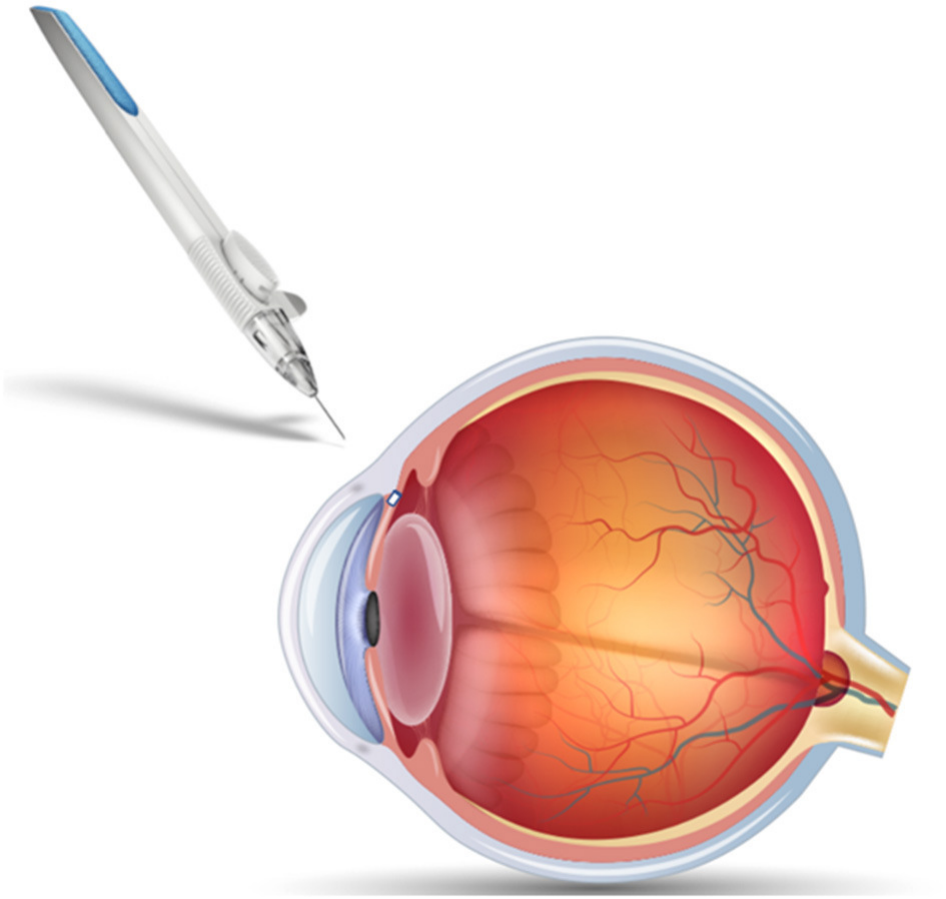
Schematic of Durysta® intracameral implant and applicator. The PLGA-based insert allows extended release of bimatoprost for treatment of open-angle glaucoma, overcoming some of the challenges associated with frequent eyedrop administration.

PGA and PLA are often co-polymerized to produce PLGA, which offers tunable monomer ratios and end groups that alter biodegradation profiles. Implementation of PLGA for ocular drug delivery vehicles is aimed at controlling the release of therapeutic through tunable polymer properties and improving biocompatibility and bioavailability. Altering the ratios of PGA and PLA also allows for optimization of degradation time, degrees of crystallinity, and hydrophobicity (44). Incorporation of a hydrophobic polymer can allow for selective permeation across mucus membranes if applied topically, or, if administered intravitreally, can work to minimize diffusion of therapeutic away from the target region and slow release. These degradation characteristics make PLGA one of the most common biomaterials found in approved polymeric ocular drug delivery vehicles. However, there have been challenges with some PLGA-based systems due to acidic degradation byproducts (44, 45). Ozurdex® is an FDA approved PLGA-based intravitreal implant used for extended release of dexamethasone in treatment of retinal vein occlusion, non-infectious posterior uveitis, and DME. The implant is co-extruded with dexamethasone to provide controlled release for 4–6 months and degrades entirely *in vivo* (46, 47). PLGA-based implant Durysta® has been recently approved for controlling IOP in patients with open angle glaucoma. A schematic of the implant applicator and location shown in Figure 2. The implant provides controlled release of the prostaglandin analog bimatoprost, lowering IOP for 4–6 months (48). Beyond these biodegradable implants, PLGA is currently being investigated for use in hydrogel, microparticle, and nanoparticle delivery systems. There have been several promising preclinical studies evaluating an intravitreal biodegradable system with PLGA microspheres embedded in a hydrogel for delivery of biologics for treatment of wet AMD (49–52). PLGA is a popular choice for ocular drug delivery due to its clinical acceptance and tunable properties, but is limited by a 6 month release duration.

PCL is composed of ε-caprolactone monomers and is formed through induced ring opening polymerization. It is a hydrophobic, semicrystalline polymer with a slow biodegradation rate, taking months to years depending on polymer molecular weight and implant size and location (53). PCL is a common choice for experimental drug delivery vehicles due to its low cost, ease of modification and copolymerization, and processability. While it has been FDA approved for other indications such as in sutures, it has not yet been approved for use in the eye (54). PCL's crystalline properties make it an excellent choice for thin films and cell delivery due to its ability to maintain structural integrity through late stages of degradation. Experimental work done by Shuamo et al. (55) and Samy (56) present PCL thin films capable of extended drug release with limited intraocular inflammation. Additionally, PCL has shown promise as an intraocular drug delivery vehicle, with recent experimental work focusing on embedding nanoparticles within contact lenses, injectable *in situ* forming hydrogels, nanoparticle emulsions and suspensions, microparticles, and capsules for treatment of several diseases including glaucoma and wet AMD (57–62).

Poly(ortho ester) (POE) is another notable polyester-based synthetic polymer that has seen use in ocular drug delivery vehicles. While POE has been FDA approved for drug delivery in other applications, it has not yet been approved for ocular drug delivery but shows potential as a promising candidate that relies on surface erosion for therapeutic release. A thermosetting PEG-Polyacetal-POE hydrogel has been developed and patented for drug delivery (63). The use of POE as a therapeutic delivery system appears to hold significant untapped potential.

### Polymethacrylates

Poly(methyl methacrylate) (PMMA) and other methacrylate-based polymer derivatives are acrylic, biocompatible thermoplastics that possess good optical clarity when processed, high mechanical strength, water transmissibility and thermal stability (64, 65). They are FDA approved for intraocular use, and were first used in ocular applications in contact lenses in 1936 (66). Methacrylate based polymeric biomaterials have expanded to include uses in nanoparticle and micelle delivery, ocular hydrogels for topical administration and intravitreal injection, and ocular implants. Notable methacrylate derivatives include poly(2-hydroxylethylmethacrylate) (HEMA) and poly(2-(dimethylamino)ethyl methacrylate) (DMAEM). Titanium-based intravitreal implant I-vation® utilized PMMA as a non-biodegradable polymer coating for sustained release of triamcinolone acetonide for patients with DME (32). The implant was successful through phase I clinical trials but was suspended in phase IIb (67). Drug eluting HEMA-based contact lenses are a growing focus in ocular drug delivery because of the large population of contact wearers. In particular, experimental work with Acuvue® HEMA-based soft contacts has shown their potential as drug delivery vehicles, capable of extended release of Ofloxacin after incorporation of a vitamin E release barrier (68). Work by Pereira-da-Mota et al. (69) utilizes HEMA and other methacrylates for experimental atorvastatin-eluting lenses for topical treatment of diabetes-related ocular diseases. The formed contacts were designed to contain molecules similar to natural cholesterol regulator 3-methylglutaryl-CoA to improve incorporation of lipophilic drugs into the polymer matrix. Experimental work with methacrylate-based polymers includes investigations into HEMA-based lenses for elution of olopatadine for allergic conjunctivitis, HEMA contact lenses with temperature triggered release for treatment of glaucoma, and DMAEM nanogel-based drug carriers to increase mucoadhesion in topically administered delivery vehicles (70–72).

### Polyolefins

Poly(acrylic acid) (PAA), also known commercially as Carbopol®, is a synthetic polymer composed of acrylic acid monomers with high water solubility and viscosity enhancing properties (73). It is biodegradable, but the resultant acrylic acid byproducts can cause inflammation (74). PAA displays good mucoadhesive properties due to its charged state at physiological conditions and has found use in several experimental hydrogel applications for anterior ocular delivery including copolymerization with PNIPAAm for generation of thermally sensitive *in situ* forming hydrogels for controlled release of epinephrine for glaucoma (75, 76). PAA is FDA approved for many topical applications including ocular administration and is commercially available in several eye drops, including the drug eluting ophthalmic emulsion Restasis®. Restasis® is a carbomer copolymer type A-based emulsion for topical administration of cyclosporine for treatment of dry eye disease (77).

### Dendrimers

Dendrimers are long branching chain polymers that have distinct physical and chemical properties depending on chain characteristics and branch functionalization. Dendrimers have found application in ocular drug delivery due to their biocompatibility, water solubility, drug entrapment capabilities, and the reactivity of terminal functional groups at the end of each branch (78). The variety of dendrimers, their ability to hold several surface charge groups, and the ease of drug entrapment make them a superior polymer system for drug delivery (79). Poly(amidoamine) (PAMAM) is a dendrimer with FDA approval for certain uses that has seen use as a tool for ocular drug delivery, but is not on the GRAS list due to toxicity concerns (80). Experimental work completed by Yavuz et al. (81). evaluated PAMAM conjugation with dexamethasone for posterior eye sustained drug release for AMD and DR while Iezzi et al. (82). investigated PAMAM conjugation with fluocinolone acetonide for treatment of retinal inflammation in AMD and retinitis pigmentosa. Both works showed successful conjugation of the therapeutic into the dendrimer and were administered *via* intravitreal injection into rats. Topical administration of PAMAM hydrogels has also been reported, with one publication presenting a crosslinked PAMAM-PEG hydrogel for controlled delivery of the anti-glaucoma drug brimonidine tartrate (83). The current regulatory status, advantages, and disadvantages of common synthetic polymers are listed in Table 1.

**Table 1:** Summary of synthetic polymers, biomaterial uses, regulatory status, benefits, and disadvantages.

**Polymer name**	**Experimental/Clinical/FDA approved biomaterial forms**	**GRAS**	**FDA approved indications**	**Approved for use in eye**	**Pros/Cons**
Poly(ethylene glycol) (PEG)	Implants, hydrogels, nanoparticles	Yes	Yes: injectables, topicals, rectal and nasal	Yes	Pros: water soluble, biocompatible Cons: fast degradation compared to other synthetic polymers
Poly(vinyl alcohol) (PVA)	Implants, hydrogels, nanoparticles	Yes	Yes: coatings, food additives, food packaging	Yes	Pros: slow degradation rate Cons: synthesized with aggressive solvents
Poly(glycolic acid) (PGA)	Implants	Yes	Yes: absorbable sutures, medical devices	Yes	Pros: fast degradation rate Cons: weak mechanical properties, brittle
Poly(glycolic acid – co – lactic acid) (PLGA)	All types	Yes	Yes: implants, drug delivery, medical devices	Yes	Pros: tunable degradation rate, water soluble, most common polymer used in ocular drug delivery Cons: acidic degradation byproducts
Poly(lactic acid) (PLA)	All types	Yes	Yes: absorbable sutures, medical devices, food packaging	Yes	Pros: Synthesized from natural sources, easily processed Cons: slow degradation rate
Poly(caprolactone) (PCL)	Hydrogels, films, nanoparticles	No	Yes: implants, delivery devices	No	Pros: easily modified, inexpensive Cons: not FDA approved for ocular applications
Poly(orthoester) (POE)	Nanoparticles	No	Yes: drug delivery	No	Pros: degrades *via* surface erosion Con: not heavily investigated for drug delivery uses
Poly(methacrylates) and derivatives (PMMA)	Hydrogels, contact lenses	Yes (mostly)	Yes: coatings, ocular lens, dental fillers, bone cement	Yes	Pros: well established ocular polymer, inexpensive Cons: non-biodegradable
Poly(acrylic acid) PAA	Hydrogels, eye drops,	No	Yes: topicals,	Yes	Pros: highly water soluble, mucoadhesive Cons: biodegradation into acidic byproducts
Poly(amidoamine) (PAMAM)	Nanoparticles, hydrogels	No	Yes: topicals, drug delivery	No	Pros: easily functionalized, contains many reactive groups Cons: not FDA approved for ocular uses

## Biopolymers or Biologically Derived Polymeric Biomaterials

**Figure 3:**
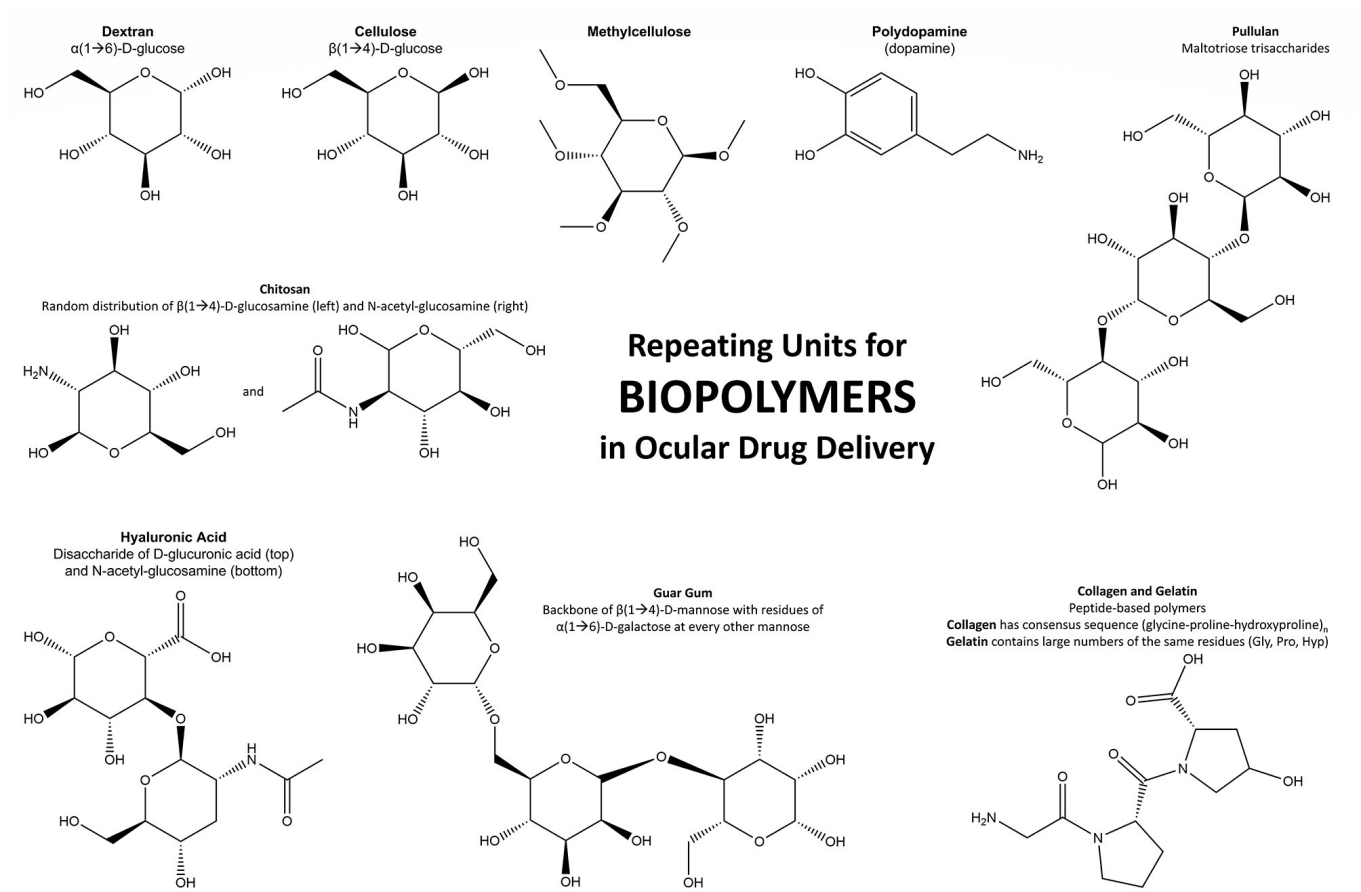
Schematic of the repeating units that form the most noteworthy biopolymers used in ocular drug delivery.

Biopolymers have become more widely used in polymeric applications as technology for production has improved and understanding of material properties increases. They are based on naturally derived monomers or building blocks (animal, plant, fungi, bacteria) and generally possess high biocompatibility, fast degradation in aqueous environments, and a broad range of viscoelastic properties with the potential to produce biomaterials for use in ocular drug delivery (84). Common biological polymers in use for ocular biomaterials and drug delivery systems include cellulose, chitosan, hyaluronic acid (HA), collagen, carboxymethyl cellulose (CMC), gelatin, dextran, guar gum, pullulan, and polydopamine. The monomers and repeating units that produce those biological polymers are displayed in Figure 3.

### Polysaccharide Biopolymers

Cellulose is considered the most common biopolymer and is derived from plant cell walls. It contains a large number of hydroxyl units and is thus very hydrophilic. It is biocompatible, biodegradable through enzymatic reactions and hydrolysis, easily conjugated and reacted, FDA approved for ocular use, and relatively inexpensive. For ocular drug delivery, carboxymethylcellulose (CMC), an ether derivative of cellulose, is the most prominent version of the polysaccharide as the addition of carboxy groups to the biopolymer chains increases water solubility (84). Due to its biocompatibility and hydrophilicity, CMC is often found in topically administered eye drops such as Refresh® or Optive® for treatment of dry eye, but many more brands and formulations are available (85). The linear nature of CMC provides an excellent framework for experimental biopolymer-based hydrogels and thin films for extended topical drug release and *in situ* forming gels for intravitreal injection. Recent work by Deng et al. (86) synthesized *in situ* forming CMC/HA hydrogels capable of releasing bovine serum albumin for up to 30 days. CMC based micro- and nano-carriers have also been produced for anterior and posterior ocular drug delivery. Experimental work from Yuan et al. (87) produced and characterized CMC-based nanowafers for extended anterior drug delivery of axitinib. The topically applied clear nanowafers contain nanoreservoirs of therapeutic for extended drug release and increased bioavailability compared to traditional eye drop delivery. Additionally, experimental CMC nanowafers for extended release of dexamethasone have been shown to effectively treat dry eye disease (88). The nanowafers contained a 500 nm array of drug reservoirs and showed successful drug release for 24 h. Another notable cellulose derivative, hydroxypropyl methylcellulose (HPMC), is commonly used in ocular drug delivery due to its viscosity enhancing properties and biocompatibility (89).

Chitosan is a polysaccharide comprised of glucosamine and N-acetyl-glucosamine monomers that possesses a strong positive charge due to primary amine groups along the backbone (90). The highly cationic nature of the polymer provides mucoadhesive benefits that have been employed for use in eye drops, to improve therapeutic bioavailability, and extended release gels for subconjunctival injection (91, 92). The amphiphilic nature of chitosan allows for improved solubility of hydrophobic drugs and increased penetration through the corneal membrane when compared to non-conjugated drug (93). Chitosan has limited FDA approval and is not currently approved for ocular applications; however, there are several publications demonstrating *in vitro* and *in vivo* efficacy (94). Technologies such as chitosan liposomes and micelles provide a high drug payload with longer drug release period that can be easily administered through intravitreal injection. Because of its cationic nature, chitosan is often employed as a polymer coating for less biocompatible anionic polymers, used in layer by layer assembly of core shell biomaterials, and used for delivery of anionic therapeutics and genetic material (60, 61, 95). Chitosan-based hydrogels have recently been investigated to increase bioavailability of the topically administered antibiotic, levofloxacin (96). Thermosensitive hexanoyl glycol chitosan hydrogels were shown to possess low ocular irritation and 1.92-fold greater bioavailability in the aqueous humor of rabbits when compared to traditional antibiotic suspension.

Like chitosan, hyaluronic acid (HA) is a hydrophilic polysaccharide made up of D-glucuronic and N-acetyl-glucosamine monomers. HA is endogenously found in many ocular tissues including the cornea, aqueous humor, vitreous humor, and retina, and fulfills a variety of important roles in the eye. HA's structure allows for high water content and potential swelling in aqueous environments and fast degradation *via* enzymatic pathways (97, 98). HA's biocompatibility, high degree of hydration, tunable water content, and viscoelastic properties have made it a popular choice for certain types of ocular drug delivery systems such as polymer gels. It has also been used as a biocompatible coating for delivery devices, and as an integral part of retinal cell based therapies (99). Work by Liu et al. (100) showed that delivery of retinal progenitor cells using a HA-based hydrogel was able to correctly transplant the cells into the sub-retinal area without disruption of function and that upon complete degradation of HA, the cells expressed mature photoreceptor markers (100). HA has also recently been applied as a self-sealing inner needle coating for intravitreal injection to minimize extraocular regurgitation of drugs (101). The most common application of HA has been as a lubricating agent in eyedrops for dry eye, with HA eyedrops serving as an artificial tear layer in products such as Optive Fusion, Vismed Multi, DROPSTAR®, Hyalistil®, and Neop (85, 102). Other applications include the Solaraze™ gel, which uses HA gel to form a depot for controlled release of diclofenac for treatment of ocular inflammation and pain (102, 103). Future research is likely to explore this further, as HA presents an easily prepared and biodegradable polymer with significant potential for the formation of degradable reservoirs for controlled drug release in addition to hydration and healing properties.

Dextran is a polysaccharide biopolymer composed of D-glucose units and is synthesized by lactic acid bacteria. It is biocompatible, biodegradable, hydrophilic, and able to form hydrogels (104). Dextran is an FDA approved biopolymer found in ophthalmic eye drop solutions such as Tears Natural Forte® and Tears Natural II® for treatment of dry eye syndrome (105). It is easily chemically crosslinked and has been experimentally investigated with chitosan, PLA/PLGA, and PEG for topical and intravitreal administration of ocular therapeutics (106). Recent experimental work has shown successful delivery of lutein, an antioxidant, from dextran-chitosan crosslinked nanoparticles for topical administration (106). Dextran is also capable of drug conjugation for ocular delivery. Low molecular weight dextran has seen experimental use as a cationic DNA carrier for targeted gene therapy to treat X-linked juvenile retinoschisis. Recent work showed successful *in vivo* transfection and expression of a dextran-protamine-DNA complex adsorbed onto the surface of solid-lipid nanoparticles after intravitreal injection into rats (107).

Guar gum is a seed-derived polysaccharide with linear backbone chains of β-d-mannose units and branch points of α-d-galactose units (108). As a biopolymer, guar gum is biocompatible, water soluble, a viscosity enhancer, capable of high degrees of swelling, mucoadhesive, non-ionic, and degradable by hydrolysis (109). The gelling ability of guar gum makes it beneficial as an additive to lubricating eye drops and is currently FDA approved for ocular use. Unfortunately, guar gum has limited solubility in alcohols and organic solvents and is unstable in solution. Derivatives such as hydroxymethyl-guar gum, hydroxypropyl-guar gum, and o-carboxymethyl o-hydroxypropyl-guar gum have been synthesized to improve solubility and stability (109). Hydroxypropyl-guar gum is found in several lubricating eye drops (110). Guar gum has been experimentally investigated to increase the bioavailability of natamycin for treatment of ocular fungal infection by integration onto PEG nanolipid carriers for controlled release from a carboxyvinyl polymer-guar gum-borate gelling system (110). Guar gum grafted PCL micelles have also been investigated for prolonged release of ofloxacin. Experimental guar gum-PCL micelles conjugated with retinol, biotinylated glutathione, and cell specific targeting agents before incorporation of ofloxacin showed drug release for at least 8 h (111).

Pullulan is a polysaccharide derived from the yeast *Aureobasidium pullulans*, composed of maltotriose units joined by α-1,6 linkages (112). Pullulan is biocompatible, non-ionic, stable over a broad temperature and pH range, water soluble, insoluble in most organic solvents, easily processed, oxygen impermeable, viscosity enhancing, and biodegradable (112, 113). Pullulan has FDA GRAS status and has been used in many experimental biopolymer applications, including ocular drug delivery (114). The non-ionic nature of pullulan often requires derivation such as sulfation or amination to incorporate a charge for improved reactivity. Co-polymerization of pullulan with other biopolymers or synthetic polymers has shown promise for extending biodegradation rate compared to the polysaccharide alone. Examples include pullulan-gellan gum electrospun nanofibers for an *in situ* forming gel for extended topical therapeutic bioavailability (115). The gelation properties of pullulan in water make it popular for use in thin films and hydrogel inserts. Experimental work completed by Pai and Reddy (116). investigated the *in vitro* properties of a 10% pullulan gel insert intended for conjunctival drug delivery. The insert showed complete degradation *in vitro* and complete drug release within 3 h of application.

### Protein Biopolymers

Collagen is a naturally occurring fibrous protein present in most connective tissue, including the cornea, sclera, lens capsule, and vitreous humor. Because it is naturally occurring, collagen is biocompatible, enzymatically degradable, can be relatively easily processed, and is widely available from primarily bovine and porcine sources. Recombinant collagen is also available, offering reduced dependence on animal sources through more consistent and safe production in plants and yeast cells (117). Collagen has a long established use in collagen shields for eye protection after ocular trauma or cataract surgery (118). More recent work utilizes collagen as a drug delivery device for encapsulated cell therapy *via* intravitreal injection, extended drug release *via* gels, or as a scaffold base for retinal tissue regeneration (119, 120). Current ocular drug delivery technologies that utilize collagen include Photrexa®, a collagen containing riboflavin ophthalmic suspension that when exposed to ultraviolet A light, crosslinks the biopolymer for treatment of progressive keratoconus (121).

Gelatin is a protein-based polymer formed from the irreversible hydrolysis of collagen. Like collagen, it is biocompatible, biodegradable, water soluble, gel forming and viscosity enhancing, readily available, and low cost, but shows advantages in lower gelation temperature and improved aqueous solubility (122, 123). It is derived from mammalian, avian, and ichthyoid collagen I sources, allowing for a broad range of available molecular weights, and is GRAS approved. Recombinant gelatin is also available to circumvent potential immunogenicity and provides access to specific gelatin molecular weights and isoelectric points (124). In ocular drug delivery, gelatin has seen applications in eye drops as a demulcent, in anteriorly and posteriorly applied hydrogels, nanoparticles for extended drug release, ocular tissue engineering, and siRNA carriers for gene therapy (105, 123, 125–127).

### Other Biopolymers

Polydopamine is a relatively recently investigated biopolymer, formed through oxidative polymerization of dopamine, one of the body's major neurotransmitters (128). Its biocompatibility and low toxicity have led to significant interest in its use in drug delivery, with particular attention paid to the development of coatings and nanostructures (128). These two applications have seen recent investigation as a novel method of ocular drug delivery. Liu et al. (129) developed an intraocular lens (IOL) with a self-polymerizing polydopamine coating capable of loading and eluting doxorubicin, preventing posterior capsule opacification (PCO) in rabbit models. Jiang et al. (130) developed polydopamine nanoparticles which showed effective loading and oxidative stress-dependent release of anti-VEGF *in vitro*. Recent findings that polydopamine coatings enhance nanoparticle mucopenetration may open the door to further applications of polydopamine in corneal drug delivery, especially as cellular uptake of these nanoparticles is also enhanced compared to uncoated nanoparticles (131). While polydopamine has only been under evaluation as a biomaterial since 2007, it has shown clear potential in ocular drug delivery, and will likely continue to mature with further research efforts (128). Several biopolymers used in ocular drug delivery are summarized in Table 2.

**Table 2:** Summary of biopolymers used in ocular drug delivery and their properties.

**Polymer name**	**Experimental/Clinical/FDA approved biomaterial uses**	**GRAS**	**FDA approved indications**	**Pros/Cons**
Cellulose	Hydrogels, films, nanoparticles, inserts	Yes	Yes: food additive, topicals	Pros: Biocompatible, nontoxic, high molecular loading potential, nanomaterial fabrication possible (132) Cons: Low solubility (132)
Chitosan	Nanoparticles, hydrogels	No	Yes: food additive, wound dressing	Pros: Mucoadhesive, positively charged at physiologic pH (133) Cons: Insoluble in neutral or alkaline solutions, brittle in hydrogel form, strong electrostatic behavior (134)
Hyaluronic acid	Hydrogels, nanoparticles, films, tissue scaffolds	No: classified as medical device currently	Yes: cosmetic fillers, injectable for osteoarthritis, topicals	Pros: Biocompatible, mucoadhesive, good viscoelastic behavior (133), naturally occurring (135) Cons: Difficult to functionalize, challenging drug conjugation (136), effect of molecular weight unclear (137)
Collagen	Hydrogels, nanoparticles, contact lenses	Yes	Yes: food additive, cosmetic injectables, wound healing	Pros: Biodegradable, biocompatible, bioactive, significant existing use in medicine (118) Cons: Immunogenicity risks, variable quality, concerns about animal sources (117)
Carboxymethylcellulose	Hydrogels, eye drops, nanoparticles	Yes	Yes: disintegrant, dental devices	Pros: Biodegradable, biocompatible, capable of sustained release, pH-sensitive (132) Cons: Challenging to develop proper viscous solutions (133)
Gelatin	Hydrogels, nanoparticles, films, tissue engineering	Yes	Yes: medical devices, food additive	Pros: Easily derived, biocompatible, rich in ECM protein, less immunogenic, transparent, low cost (123) Cons: Strength depends on source and processing conditions, still immunogenic, challenging to safely crosslink (123)
Dextran	Hydrogels, films, nanoparticles	Yes	Yes: shock and other blood related indications, inhalant	Pros: Excellent biocompatibility (134) Cons: Difficult to functionalize (134)
Guar Gum	Hydrogels, films	Yes	Yes: food additive	Pros: Mucoadhesive, antioxidant, low antigenicity, biodegradable, low cost, stable and biocompatible (138) Cons: Brittle, high swelling, poor mechanical properties in hydrogel, low recoverability (138)
Pullulan	Hydrogels, nanoparticles, eye drops, fibers	Yes	Yes: food additives, tablet coatings, stabilizer, and thickener	Pros: Easily derived, stable, good film-forming properties, biodegradable (116), nontoxic (139) Cons: Unexpectedly slow diffusion (140), requires functionalization to load drugs (141)
Polydopamine	Nanoparticles, Intraocular Lenses	Not evaluated	No: Dopamine HCl indicated for correction of hemodynamic imbalances	Pros: Biocompatible, biodegradable, low toxicity, excellent adhesion Cons: Synthesis is challenging to control and poorly understood, need for investigation of toxicology, degradation, elimination

## Polymeric Biomaterial Forms

### Micro- and Nano-Scale Technologies

**Figure 4:**
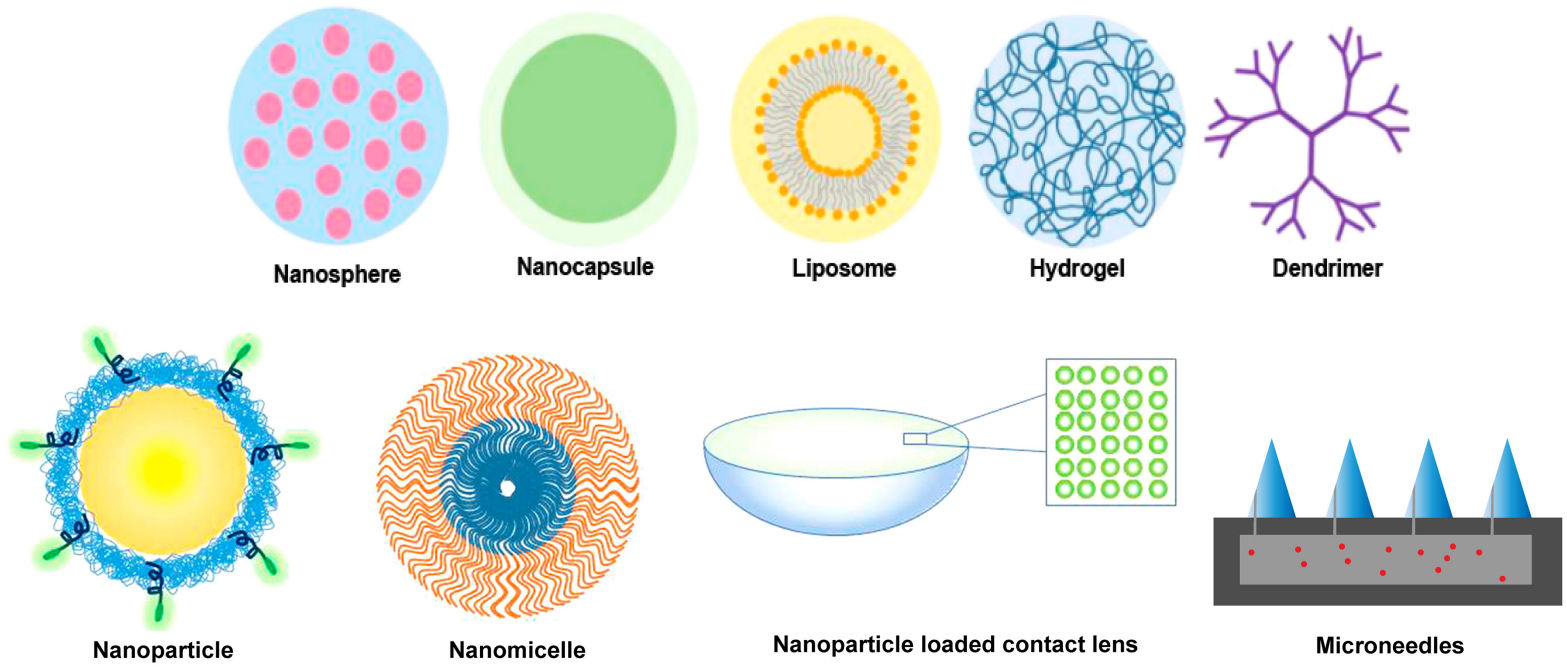
Various polymer forms that have been applied to facilitate and modulate ocular drug delivery at the macroscale and nanoscale. Both synthetic and biopolymers can be formulated into nanospheres, nanocapsules, liposomes, hydrogels, dendrimers, nanoparticles, nanomicelles, and microneedles. Nanoscale polymers can be incorporated into composites, such as the hydrogel-based contact lens shown with nanoparticles.

The versatility of biopolymers and synthetic polymers opens the door to many types and forms of biomaterials used as drug delivery vehicles to treat ocular diseases. Within the field of micro- and nanotechnology, there are a variety of drug delivery vehicles such as microparticles, nanoparticles, micelles, and liposomes. These drug delivery vehicles show significant promise in the eye due to their less invasive application approaches topically as well as ease of injection through small gauge needles (44, 142). These have also been explored for incorporation into drug-eluting contact lenses to facilitate topical delivery (143). Figure 4 presents several ocular drug delivery forms that utilize nanotechnology.

Microparticles are small-scale particles generally in the size range of 1–1,000 μm. Microparticles have been evaluated for ocular drug delivery for decades, and typically demonstrate higher drug loading capacity and release duration than nanoparticles due to the larger size of the particles, but a balance between drug loading and size considerations for injectability must be established. Several articles have focused on microparticles in the range of 1–50 μm for intravitreal injection to balance these considerations (144). It has been recently proposed to use nanoparticles embedded in microparticles to overcome some of these challenges (145). Microparticles have also shown controlled variable monodispersity upon application, demonstrating versatility of this approach.

Nanoparticles are particles between 10 and 1,000 nm which can possess a surface charge, based on monomer properties, that allows for increased permeability or mucoadhesion of the therapeutic (146). Nanoparticles allow for drug delivery through encapsulation of the target therapeutic or surface loading through electrostatic interactions. Most of the biopolymers and synthetic polymers discussed in this review have been prepared as nanoparticles and extensively evaluated for drug delivery from contact lenses, intravitreal injection, topical, and suprachoroidal administration (144, 147, 148). Nanoparticles have the advantage of being small enough to penetrate cells, maximizing therapeutic efficacy through targeted therapeutic release. Their small size also facilitates overcoming many of the barriers to ocular delivery. While there are many advantages to nanoparticles and there has been a significant shift to focus on nanoparticles for ocular drug delivery in recent years, a nanoparticle ocular drug delivery system has yet to be commercialized (149).

Experimental systems include GB-102®, a PLGA microparticle-based drug delivery vehicle designed by Graybug Vision for treatment of wet AMD and macular edema. The injectable drug depot is currently in clinical trials and has shown controlled release of sunitinib malate for up to 6 months post injection (32). POE-based nanoparticles maintained vitreous localization in rabbits after intravitreal injection for up to 14 days with minimal increases in IOP (150). Work by Fu et al. (151) developed poly(ortho ester urethane) nanoparticles that showed pH-sensitive degradation properties and were efficient in delivering doxorubicin to *in vitro* tumor spheroids. Experimental work by Jiang et al. (60) utilized chitosan's cationic properties for intravitreal delivery of anti-VEGF bevacizumab through microparticles composed of a cationic chitosan core and PCL shell. Chitosan nanoparticles have also been evaluated for transscleral delivery of bevacizumab (152). Lu et al. reported bevacizumab-loaded chitosan nanoparticles for treating DR (153). Work by Dionisio et al. (154) modified pullulan through sulfation and amination to produce both negatively and positively charged pullulan-chitosan and pullulan-carrageenan nanoparticles for transmucosal drug delivery, while Garhwal et al. (155) integrated experimental pullulan-PCL nanospheres encapsulating ciprofloxacin into HEMA-based contact lenses for topical administration of antibiotics. Recent research on corneal applications of gelatin include positively charged gelatin nanoparticles for extended release of moxifloxacin (156). The particles showed *in vitro* drug release up to 12 h and showed *in vivo* antimicrobial properties superior to the market available product MoxiGram®.

Micelles are core/shell structures formed by amphiphilic block copolymers with hydrophobic and hydrophilic domains, and are often <100 nm in size. Polymeric micelles can enhance solubility of poorly soluble drugs and are being explored for use in promoting drug transport through the cornea and sclera (157). Micelles offer several advantages to enhance topical delivery, including thermodynamic stability, relative ease of preparation, high loading capacity, and lack of interference with optical properties of devices or solutions (143). These are likely to be adopted clinically due to relatively simple and inexpensive fabrication techniques (158). Micelles have been explored for several classes of therapeutics including cyclosporine, anti-inflammatories, immunosuppressants, anti-glaucoma drugs, antifungals, antivirals, and experimental antioxidants (159). Several stimuli-responsive poloxamers have been evaluated, including PF-127 for topical delivery of a hydrophobic drug to the anterior segment for treatment of allergic conjunctivitis (160), PF-68 for delivery of ferulic acid (161) or enhancing solubility of gatifloxacin in contact lenses (162), and their combination for delivery of antifungals (163). Triamcinolone acetonide delivery with PEG-block-PCL and PEG-block-PLA micelles was also evaluated (164). Other types of polymeric micelles evaluated include amino-terminated PEG-block-PLA and HPMC for delivery of tacrolimus (165). Chitosan has even been explored for micellar delivery (166), including delivery of dexamethasone (167), and HA has been conjugated to peptides to enhance solubility through micelles (97). Challenges that remain include improving micelle stability for longer shelf-life and therapeutic delivery duration. These factors can be controlled by polymer molecular weight, hydrophobicity/hydrophilicity, and block arrangement, with promise demonstrated with pentablock copolymer micelles (168). Further, micelles can be assembled into larger hydrogels to extend delivery (58).

While liposomes are not polymers, they have been used with polymers for ocular drug delivery. Liposomes have a cell membrane-like structure made from one or more phospholipid layers, enabling adhesion to cell membranes. They can be complexed with polymers to facilitate ocular drug delivery by improving liposome stability (148). Liposome conjugates evaluated for ocular drug release have included chitosan, silk fibroin, and PEG (169, 170).

These small systems have several advantages, including ease of injection, extended topical release, and enhanced permeability. Two key challenges are establishing long-term extended release and increasing drug loading efficiency. Other challenges include preserving therapeutic activity during preparation and loading of these delivery systems. That being said, injecting a micro- or nano-delivery system 2–3 times per year may still be a viable option for patients receiving more frequent intravitreal injections since injection would still be in office through a small gauge needle.

### Hydrogels

Hydrogels consist of bound networks of synthetic and/or biologic hydrophilic polymers that absorb aqueous fluids. Many hydrogels exist specifically for intra- and extra-ocular applications ranging from contact lenses to vitreous substitutes. Hydrogels have also been used for drug delivery and/or cell encapsulation, and have been evaluated for use with optic nerve stabilization (171) and intraocular lens prosthetics (172). The large array of ocular applications may be attributed to both the hydrophilicity of hydrogels and the customizability of component polymers.

Hydrogels possess several modifiable and customizable properties such as degree of swelling, biodegradability/erosion, viscoelasticity, pore size, diffusion rate/flux, permeability, stimuli responsiveness, and drug/protein loading efficiency. Each of these characteristics may be directly or indirectly impacted by changing one or more of the following: degree of crosslinking (crosslinking density), monomer and polymer concentrations, and polymer type (atomic/molecular composition). The inherent hydrophilicity of hydrogels can provide systems with biological and mechanical stability in various ocular environments. A hydrogel's degree of crosslinking and hydrophilicity have a strong impact on the degree of swelling by their effect on variables such as pore size and diffusion rate/flux, which can be used to tune drug release. The aqueous environment in hydrogels allows investigators to mimic the extracellular matrix and tissues for cell delivery systems, may provide stability and improve cellular uptake for hydrophilic drugs, genes, and biologics. However, some therapeutics suffer reduced bioactivity in aqueous environments, and modifications may need to be made to incorporate hydrophobic drugs or prevent fast elution of hydrophilic drugs.

Due to the customizable nature of hydrogels and vast array of viable polymers, this area of research has potential for clinical translation and continued development. One specific area of recent growth for ocular applications is *in situ* hydrogel formation, where a hydrogel undergoes gelation (polymerization and/or crosslinking or self-assembly) in the ocular environment in response to specific stimuli (light, temperature, pH, oxygenation, etc.). From intraocular applications such as intravitreal injections to topical treatments with films and inserts, hydrogels formed *in situ* show promise as a major player in the future of ocular drug delivery. *In situ* forming gels enable injection through smaller gauge needles, facilitating intraocular delivery in an outpatient setting. Furthermore, *in situ* formation can enable conformal coating of curved surfaces like the cornea, enabling direct contact and more consistent drug delivery.

Xie et al. (173) developed a nanoparticle/hydrogel composite for the sustained release of an anti-tumor therapeutic to the posterior segment of the eye. The hydrogel, composed of collagen II and sodium hyaluronate, was formed *in situ* following injection in to the vitreous and in response to physiological temperature stimuli. Thermo-responsivity was attributed to a thermo-responsive crosslinking reaction at 37°C between amine groups of collagen and succinimidyl groups of the additive 8-arm PEG succinimidyl glutarate (tipentaerythritol). Another injectable hydrogel was presented by Osswald et al. (51) to deliver anti-VEGF to the choroid *via* intravitreal injection. This hydrogel consisted of poly(N-isopropylacrylamide) (PNIPAAm) and poly(ethylene glycol) diacrylate (PEGDA) and utilized the properties of PNIPAAm to create a thermo-responsive *in situ* forming hydrogel. In 2016, researchers developed a hydrogel that underwent gelation upon exposure to aqueous conditions (174). This unique *in situ* gelation method was the product of hydrophobic interactions between poly(ethylene glycol) methacrylate (PEGMA) and vitamin E methacrylate leading to the formation of physical crosslinks. This hydrogel's chemistry and crosslinking ability has potential in generating hydrogels capable of delivery of hydrophobic drugs.

Drug delivery coordinated with tissue replacement, such as intraocular lens implantation and vitreous substitution, is a relatively recent area of research. This work shows great promise by potentially offering a reduction in frequent administration or procedures and mitigation of post-operative complications. Tram et al. (175) proposed a solution for combatting cataract formation following vitrectomy by loading PEG-based hydrogel vitreous substitutes with the antioxidant ascorbic acid. Building off of that research, they found that glutathione may be a useful addition to ascorbic acid in ocular drug delivery (13). Polymer coatings for IOLs, made of polydopamine or synthetic polymers, are being evaluated to reduce complications after cataract surgery from infection and PCO (129, 176).

While significantly less invasive than injections and tissue replacement strategies, topical hydrogel drug delivery solutions present their own challenges, requiring prolonged contact with tissues of interest and firm shape retention. One example of a topical *in situ* forming hydrogel was reported by Anumolu et al. (177) to deliver doxycycline to the cornea using PEG octamer hydrogels (177). The hydrogels were pH-responsive, undergoing shape-retaining gelation within seconds of application. Another example of a viable *in situ* forming hydrogel used for sustained drug delivery was recently published by El-Feky et al. (178), who installed the gel into the inferior conjunctival fornix to deliver nifedipine for glaucoma treatment (178). Hydrogels were created using poloxamer 407 (P407) and HPMC, utilizing properties of P407 to incorporate thermo-responsiveness into the hydrogels. Fedorchak et al. (179) made use of a thermo-responsive PNIPAAm hydrogel eyedrop containing drug-loaded PLGA microspheres to achieve long-term delivery of brimonidine for glaucoma treatment, showing efficacy in rabbit models out to 28 days after administration.

*In situ* gelation provides a drug delivery solution that is tailored to the patient's ocular geometry and has great potential in reducing both treatment frequency and procedure invasiveness. Opportunities for innovative hydrogel solutions for ocular drug delivery are ever-growing, opening doors for many more future research projects and likely commercial translation in the near future.

### Fibers, Films, Rods, Extrusions

Processing polymers into fibers, films, rods, or extruded forms allows various alternative configurations for drug delivery systems. These delivery methods and geometries may even be interconnected. For example, fibers may be formed *via* electrospinning to create a rod-shaped implant, or the fibers may be spun into a sheet and hydrated to form a film.

Kelley et al. (180) detailed and compared production methods for hot-melt extrusion manufacturing of dexamethasone-loaded injectable intravitreal implants. The extruded rods were composed of PLGA with varying weight percentages of acid- and ester-terminated PLGA to control the implant degradation and drug release rate. OZURDEX® (Allergan) is an FDA-approved intravitreal implant that employs extruded PLGA (NOVADUR® technology) for sustained dexamethasone release through biodegradation (181).

One method for producing fibers is electrospinning. A recent study experimented with various configurations for conjunctival fornix inserts for sustained delivery of besifloxacin to the cornea for treatment of bacterial keratitis (182). The inserts, synthesized *via* electrospinning, were prepared as fibers of PCL and PEG and then coated with biopolymers—either sodium alginate or thiolated sodium alginate—to confer mucoadhesion. Another ocular insert composed of electrospun PCL and intended for insertion into the conjunctival fornix was developed to deliver fluocinolone acetonide to the retina and was evaluated in pre-clinical studies (183). PCL and chitosan capsules have also been prepared *via* electrospinning to fabricate a hollow bilayered design for intravitreal injection (61). Delivery systems designed with electrospun nanofibers present two specific advantages: tunable device porosity for controlled drug diffusion and a high ratio of surface area to volume for increased chemoadsorption (61).

Electrospun conjunctival fornix inserts were also investigated for the delivery of triamcinolone acetonide to the anterior and superficial segments of the eye (184). Investigators analyzed properties and release profiles of nanofiber formulations with varying concentrations of PVA, poly(vinylpyrrolidone) (PVP), zein/eudragit, and chitosan, identifying an optimal formulation of only PVP and chitosan.

Electrospinning has also been applied to develop both *in situ*-forming and pre-hydrated hydrogel systems. Göttel et al. (139) presented an *in situ* gelation system that begins as a curved lens of electrospun pullulan and gellan gum fibers. Upon application to the ocular surface, the fibrous lens hydrates to form a film/hydrogel. A different study utilized electrospun PVP and HA nanofibers to develop hydrogels for drug delivery (185). This study focused on developing an ocular insert to deliver ferulic acid and Epsiliseen®-H for treating ocular surface conditions. PVP was employed to enable electrospinning of HA while HA was the polymer responsible for the drug delivery mechanism.

Films are comparable to hydrogels for drug delivery as they hydrate to form an aqueous system. They also show potential in drug delivery, particularly for topical applications. A porous resorbable film was recently investigated as a bandage contact lens following corneal injury (186). The films were composed of bovine serum albumin (BSA) structural nanofibrils and the antioxidant kaempferol.

One recent advancement in fiber and film technology is the PRINT® technique. This technology allows researchers to precisely control a film's shape, size, surface properties/functionality, chemical composition, porosity, and moduli (187, 188). Researchers have employed PRINT® to customize drug delivery systems on the nanoscale-level to control for release profiling/kinetics and environment of application (189). The technology can use an array of biopolymers and therapeutics including peptides, nucleic acids, and antibodies (146, 187). PRINT® has been used to develop subconjunctival implants, intracameral implants, intravitreal implants, nano-and micro-suspensions, etc (190). One recent development with PRINT® technology is the AR13503 (Aerie Pharmaceuticals) implant, which utilizes PLGA, PDLA, and PEA to control delivery to the retina for more than 2 months and is in phase 1 clinical trials (190–193). Another delivery system developed with PRINT® is an Envisia Therapeutics implant (ENV515) currently in phase 2 clinical trials (194). Results thus far suggest that ENV515 is effective in lowering IOP for 28 days (195, 196). Results from a 12-month study found 25% IOP reduction compared to topical ophthalmic solution (195). PRINT® shows great promise for its ability to customize polymer-based ocular drug delivery systems at the nanoscale level.

Polymer processing techniques are well developed in other applications and are beginning to emerge in ocular drug delivery systems. These processing techniques will be required for manufacturing of several ocular drug delivery devices and give potential to explore innovative new delivery systems using already approved polymers.

## Administration Methods

### Eyedrop Delivery

Eyedrops have seen widespread usage for delivering a variety of medications for ocular disorders, thanks to their ease of use, low cost, and relatively good patient compliance (142, 197). However, in recent years, their limitations as a drug delivery system have led to significant research effort invested in improving their capacity or developing more efficient alternatives (198). While eyedrops offer excellent delivery efficiency for topical diseases of the eye, their efficiency significantly declines when used to deliver pharmacologic agents to certain tissues in the eye. By some estimates, as little as 1–5% of an eyedrop's drug content can reach the anterior segment of the eye due to the barriers it faces in passing through the cornea to the aqueous humor (142, 158, 199). First among these is the rapid turnover of the tear film on the cornea, which leads to a significant fraction the eyedrop's volume following the tear film into nasolacrimal drainage and systemic circulation (200–202). By some estimates, as much as 80% of the tear film's volume can be turned over in a minute when eyedrops are applied to the eye, leading to significant loss of dose volume (200). This lost drug dosage then enters systemic circulation, where it may be metabolized before reaching ocular tissue and risks triggering systemic side effects that compromise patient health (202). Any drug not cleared *via* tear film drainage must still penetrate corneal tissue in order to reach the anterior chamber and have a therapeutic effect on ocular tissue. The structure of corneal tissue makes it difficult for both hydrophilic and lipophilic molecules to pass through. The corneal epithelium admits only lipophilic drugs smaller than 10 Å through cell-mediated transport mechanisms, and forces hydrophilic drugs to diffuse through paracellular pathways blocked by tight junctions (19, 158). The corneal stroma, meanwhile, is highly hydrophilic, slowing the movement of the lipophilic drugs that pass the epithelium while allowing freer movement of the few hydrophilic molecules that enter (158).

Despite these challenges to drug retention and penetration, eyedrops are still favored for the treatment of diseases in the anterior segment of the eye. Their ease of delivery has also made them attractive for delivery to the posterior of the eye, with researchers investigating a variety of eyedrop formulations with improved drug retention and penetration characteristics, with some working toward eye drop formulations for posterior ocular delivery to overcome the limits of injections (201, 203–207).

The combination of rapid clearance and the extreme difficulty of corneal penetration has led to significant research efforts aimed at increasing the delivery efficiency of eyedrops. One of the earliest options explored was to simply increase the concentration of drug delivered in the eyedrop solution, overcoming delivery barriers through essentially brute force. However, this option presents its own challenges, as such high drug doses and accompanying polymer and preservative exposure could cause local irritation or toxicity in patients (207–210). In addition, the higher drug dose per eyedrop leads to higher doses draining to the bloodstream, potentially exacerbating systemic side effects (208). As an alternative to increasing dose per eyedrop, some medications instead recommend increasing the frequency of eyedrop administration. However, this presents its own challenges, as higher frequency administration has been linked to significant reductions in patient compliance with treatment regimens (207, 211). Patients with physical or visual impairments, as well as children who are unable to administer eyedrops to themselves, may be especially non-compliant, as eyedrops rely on self-application to have an effect (210). In addition, frequent repeated application of eyedrops may still lead to local and systemic side effects associated with high dosing (207).

Because of these continued challenges in increasing delivery efficiency of eyedrops, modern research has investigated a variety of polymer-based solutions for enhancing drug penetration and residence time in the anterior eye. One solution is the development of polymer nanocarriers with mucoadhesive capabilities. These nanoparticles can entrap themselves in the mucus layer that covers the cornea, with some even capable of penetrating corneal tissue to enter the aqueous humor thanks to their small size (158, 200, 202, 205). Mucoadhesion lengthens the residence time of drug delivery systems significantly, allowing them to more effectively release their drug payload for uptake by ocular tissue. Corneal penetration is an even more desirable outcome, as the ability to effectively penetrate the cornea using a drug carrier provides immense opportunities for delivery to intraocular spaces. Recent research efforts have developed chitosan and PLGA nanoparticles capable of reaching the retinal surface, a demonstration of how nanoparticles can help solve the challenge of developing eyedrops capable of posterior ocular delivery (211, 212). Another option is the addition of polymer viscosity enhancers and gelling agents such as xanthan gum, which increase the residence time of an eyedrop atop the cornea, thereby giving more time for the drug payload to begin penetrating corneal tissue (19, 213). Both of these solutions make use of a variety of polymers. While they still face significant challenges in successful implementation and translation from laboratory to clinical use, several preclinical studies are making use of gelling systems to improve drug delivery efficiency through eyedrops. One interesting recent development has been investigation into thermosensitive polymers that form gels at physiologic temperatures (207, 214). These polymers could allow future eyedrops to be administered in solution at room temperature, then form a hydrogel reservoir on contact with the warmer tissue of the eye, providing an easily administered long-lasting form of ocular drug delivery.

### Subconjunctival Injections and Implants

Injection of pharmacologic agents presents an attractive alternative route for the delivery of drugs to ocular tissue. Injection into the subconjunctival space specifically allows drugs to be released next to the sclera and avoid corneal barriers to entry (202). Drugs are able to easily penetrate the more permeable scleral layer, potentially enabling significantly more efficient delivery to the interior of the eye, particularly the posterior segment (197, 201, 209, 213). While subconjunctival drug injections and implants necessitate a relatively more invasive procedure than eyedrops, they offer the potential of prolonged drug delivery compared to eyedrops, potentially lasting months between injections or implant replacements (19, 199). This would represent a significant advantage in patient compliance, as a minimally invasive injection or implantation procedure every few months is significantly easier to maintain compared to daily eyedrop administration regimens (19, 158). This method is not without challenges, however, as the subconjunctival space, while not as severely drained as the anterior surface of the eye, is still rich in drainage routes. Conjunctival and scleral blood vessels, as well as lymphatic drainage, will interfere with delivery and cause some of the administered dose to enter systemic circulation rather than penetrate the sclera and enter the eye (201, 202). In addition, the choroidal tissue in the eye poses an additional barrier to lipophilic drug delivery, as this tissue can bind lipophilic drugs (213).

The significant potential of subconjunctival delivery to bypass the challenges of eyedrop administration in a minimally invasive manner has led to research efforts focused on overcoming the challenges of clearance and penetration while extending the duration of drug release after implantation or injection. Polymer solutions for these problems include polymer micro- and nano-particles which, similar to their role in eyedrop formulations, help improve drug residence time near ocular tissue and assist in penetrating the scleral barriers to ocular entry, thereby increasing the drug dose delivered (158, 201, 202). Alternatively, subconjunctival injections of drug-loaded hydrogels composed of polymers such as PEG, PLGA, and HA can create a reservoir capable of extended release over a course of weeks or months, offering a more easily prepared alternative to micro- and nanoparticle systems (197, 208). Finally, polymeric subconjunctival implants offer a more stable platform for drug delivery through the subconjunctival space, with research publications describing devices made of PDMS, PLGA, and polyurethane among others (19, 197, 209). Animal studies into the use polymer-based subconjunctival drug delivery systems have found promising initial data, with favorable biocompatibility and safety results for polyimide and PLGA implants and evidence of extended-release efficacy for PLGA microspheres in the subconjunctival space (215, 216). Further research into delivery through the subconjunctival space is likely to offer significant potential for improvement of drug delivery compliance and outcomes. Many of these research efforts may benefit from prior developments in subconjunctival drainage devices designed to relieve IOP and assist in glaucoma treatment, as numerous polymer drains have already received approval for market use (217).

### Suprachoroidal Injections

Another alternative route for drug delivery is injection to the suprachoroidal space, a thin layer of tissue between the sclera and choroid of the eye (218). In theory, injections into this space could quickly spread across the inner surface of the eye, allowing rapid access to the posterior tissues of the eye with limited loss to the vitreous humor (201, 218). This would provide a highly specific pathway for delivery to these tissues with minimized adverse effects from off-target delivery and significantly lower invasiveness compared to intravitreal injection (213). However, the suprachoroidal space is extremely delicate, with only 30 μm of tissue thickness in the region and a recommended maximum injection volume of only 200 μl (218). Higher volumes than this risk causing choroidal edema and detachment (218). In addition, as this space has been relatively underexplored, there is a significant chance that yet-undiscovered safety challenges may emerge with the use of a broader range of polymers and injection systems. Perhaps because of these significant challenges to safe and accurate delivery, there has been relatively minimal exploration and characterization of the suprachoroidal space, with early studies beginning only in the mid-2000s and testing of suprachoroidal delivery in animal models of ocular disease by the early 2010s (219, 220).

Einmahl et al. investigated the suprachoroidal space's tolerance of POE in rabbit models, finding no evidence of complications or intolerance over the 3 weeks the polymer remained in the space (221). In recent years, microneedle-based injections to deliver drug-laden solutions into the suprachoroidal space have been frequently explored, as they are a minimally invasive method with less risk of complications and rapid sealing of the injection site (201). Polymers investigated in these suprachoroidal microneedle injections serve a variety of roles, from simple injection excipients to the focal point for investigation. Chiang et al. (222) used injections of CMC to form hydrogels capable of swelling after injection to evaluate their effect on the thickness of the suprachoroidal space. They also explored the use of polymers as injectable drug delivery excipients by evaluating the distribution of FITC-labeled CMC and HA in the suprachoroidal space following microneedle injection (223). One possible innovation in this area is the use of PRINT® technology, which has been previously used to produce microneedle arrays for transdermal drug delivery (224). This application of PRINT® has been licensed for use by Aerie Pharmaceuticals and may be employed for suprachoroidal microneedle systems in the future (189). Jung et al. (225) investigated the utility of HA hydrogels as a means of directing suprachoroidal drug delivery, using the hydrogel's swelling behavior to push drug-laden polymer microparticles toward the posterior ocular tissue. These investigations demonstrate novel potential applications of polymers in ocular drug delivery and may provide a foundation for future innovation in suprachoroidal delivery.

### Intravitreal Injections and Implants

While subconjunctival and suprachoroidal injections and implants offer a more efficient alternative to eyedrops for drug delivery to the eye and are more effective at both anterior and posterior delivery, they are still subject to limitations due to the tissue and drainage barriers they face when releasing drugs (201). Delivery directly to the vitreous humor bypasses corneal and scleral tissue barriers and ensures high drug delivery efficiency, drug bioavailability, and precise control of therapeutic concentrations, especially to tissues in the posterior eye (20, 142, 200, 213). For this reason, in spite of its invasive nature, intravitreal injections are currently a popular choice for drug delivery to the posterior segment. However, injections of drug solution without controlled release systems still face rapid clearance in the vitreous, necessitating frequent injections to maintain therapeutic levels of drug in the eye (213, 226). This is problematic for patients, as this procedure requires ophthalmologists to administer the injections and risks significant side effects. These range from more manageable issues, such as elevated IOP and endophthalmitis, to severe and potentially vision-altering side effects such as retinal detachment and intravitreal or subconjunctival hemorrhage (142, 197, 201, 203). In addition, drug that has been injected must still contend with diffusion through the vitreous humor to reach target tissues, a process made more difficult by rapid clearance due to vitreal circulation, the charge of vitreal fluid interfering with the diffusion of charged molecules, and the vitreous humor's extracellular matrix hampering large molecule movement (197, 227). While this method does offer some advantages over topical and subconjunctival delivery, these challenges limit its effectiveness in current drug delivery applications.

**Figure 5:**
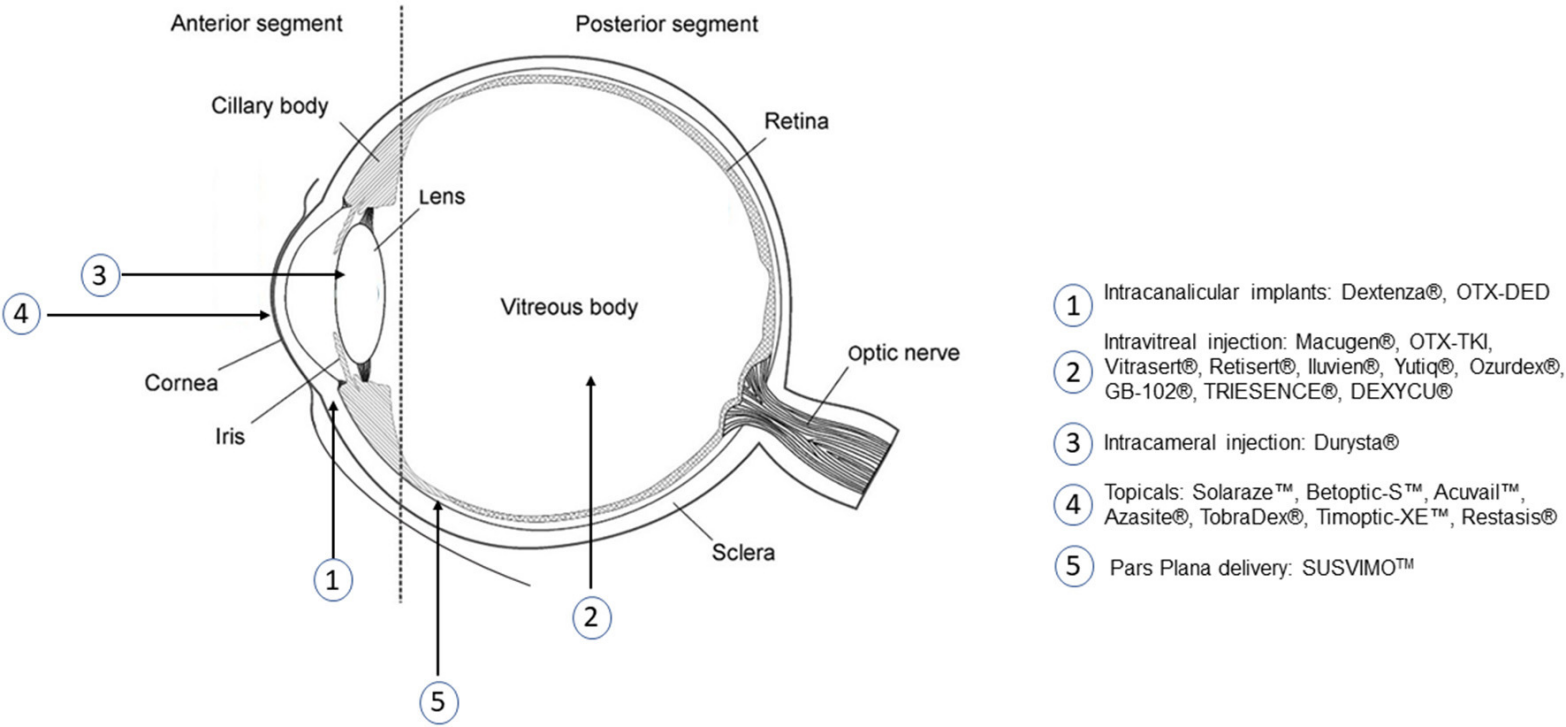
Administration location of several FDA approved ocular drug delivery systems that use polymers.

To overcome these challenges, significant effort has been invested in the development of intravitreal drug delivery systems. Recent examples include a thermoresponsive polymer made of a combination of pentaerythritol, lactic acid, and ε-caprolactone functionalized with PEG and another thermoresponsive hydrogel made of PEG-poly(serinol hexamethylene urethane), which can be injected into the intraocular space to serve as a controlled-release system for extended drug delivery (226, 228). Researchers have also investigated a variety of polymer nanoparticles, using materials such as PCL and PLGA to develop drug-loaded nanoparticles for intravitreal injection (226, 227). Others have developed intravitreal implants out of materials such as PLGA, silicone, polyimide, and PVA. The goals of these systems are to increase the duration of drug release, thereby reducing injection frequency and its associated risks without exposing the eye to additional risks from the polymers themselves. This is a delicate balance, which will require significant research effort to maintain, but the potential benefits of an extended-release intravitreal drug delivery system are highly promising. Several labs are investigating additional polymer systems for intravitreal use. This includes our work developing polydopamine nanoparticles for anti-VEGF delivery, as well as efforts by other labs developing technologies such as phase-inversion mixtures of polymer and solvent, PEGylated siloxanes, and NIPAAm-based thermoresponsive polymers for intravitreal (130, 229, 230). Some of these systems are currently being translated for commercial use, such as ReVana Therapeutics' EyeLief™ and OcuLief™ injectable polymers (231). One system with particularly promising results is the Genentech Port Delivery System, SUSVIMO^TM^ a reloadable port composed of a polysulfone body coated in silicone, which recently received FDA approval for delivery of ranibizumab for the treatment of wet AMD (232, 233). Figure 5 contains a schematic of some of the FDA approved polymeric biomaterial products and administration location.

## Regulatory Status of Ocular Drug Delivery Systems

While there is significant effort being invested in the development of polymer-based ocular drug delivery systems, a key challenge is the translation of these systems to clinical use. A number of products have successfully reached the market over the last few decades, with all four administration methods discussed previously having at least one FDA-approved drug delivery system that includes polymers to enhance their function. Notable examples are shown in Table 3. Eyedrops, the most mature drug delivery platform of the four, understandably have a significant number of polymer products, with numerous formulations approved for the treatment of diseases such as glaucoma, bacterial conjunctivitis, and uveitis (200, 213). Most make use of these polymers to increase the drop's residence time and release efficiency. Other applications such as polymer nanocarriers and thermosetting gels are still under investigation to evaluate their utility in extending the duration of eyedrop drug release and drug penetration (200, 202). Research into using eye drops for posterior segment delivery could have significant implications in the field of ocular drug delivery.

**Table 3:** Polymer-based ocular drug delivery systems with FDA approval.

**Product**	**Polymer(s)**	**Pharmaceutical**	**Delivery route**	**Indication**
TRIESENCE ® (234)	Carboxymethylcellulose	Triamcinolone Acetonide	Intravitreal Injection	Uveitis, temporal arteritis, sympathetic ophthalmia
Iluvien® (235)	Polyimide/Silicone/PVA	Fluocinolone Acetonide	Intravitreal Implant	Diabetic macular edema, uveitis
Ozurdex® (236)	PLGA	Dexamethasone	Intravitreal Implant	Diabetic macular edema, macular edema, uveitis
Retisert® (46, 237)	Silicone/PVA	Fluocinolone Acetonide	Intravitreal Implant	Posterior uveitis
Yutiq® (46)	Polyimide	Fluocinolone Acetonide	Intravitreal Implant	Posterior uveitis
Vitrasert (200)	PVA/Ethyl Vinyl Acetate	Ganciclovir	Intravitreal Implant	Retinitis
Dextenza (238)	PEG-Fluorescein	Dexamethasone	Intravitreal Implant	Postsurgical ocular inflammation
DEXYCU® (46)	Acetyl triethyl citrate	Dexamethasone	Intravitreal Implant	Postsurgical ocular inflammation
Durysta™ (48)	PLGA/PGA/PEG	Bimatoprost	Intravitreal Implant	Open angle glaucoma, ocular hypertension
Macugen® (21)	PEG	Pegaptanib sodium	Intravitreal Injection	Late stage AMD
SUSVIMO™ (232, 233)	Polysulfone/Silicone	Ranibizumab	Refillable Intraocular Implant	Wet AMD
ACUVUE® and others (239)	PHEMA, PMMA	None (contact lenses)	Anterior eye placement	Vision correction
Optive (85)	CMC	CMC	Eyedrop	Dry Eye Syndrome
Optive Fusion (85)	HA/CMC	HA/CMC	Eyedrop	Dry Eye Syndrome
Restasis® (77)	PAA	Cyclosporine	Eyedrop	Dry Eye Syndrome
Neopt (85)	HA	HA	Eyedrop	Dry Eye Syndrome
Vismed Multi (85)	HA	HA	Eyedrop	Dry Eye Syndrome
Tears Naturale® (240)	HPMC, Dextran 70	HPMC	Eyedrop	Dry Eye Syndrome
GenTeal® (240)	HPMC	HPMC	Eyedrop	Dry Eye Syndrome
Betoptic-S™ (200)	Polystyrene-divinylbenzene	Betaxolol	Eyedrop	Open angle Glaucoma
Acuvail™ (200)	CMC	Ketorolac Tromethamine	Eyedrop	Postsurgical ocular pain and inflammation
Azasite® (200)	Polycarbophil	Azithromycin	Eyedrop	Bacterial conjunctivitis
Lacrisert® (200)	HPMC	None (ocular insert)	Anterior eye placement	Dry Eye Syndrome
Mydriasert (200, 241)	Ammoniomethacrylate polymer	Tropricamide, phenylephrine HCl	Anterior eye placement	Induced preoperative mydriasis
TobraDex® (200, 242)	Hydroxyethylcellulose	Tobramycin, Dexamethasone	Eyedrop	Blepharitis
Timoptic-XE™ (200)	Gellan Gum	Timolol maleate	Eyedrop	Glaucoma
Xen Gel (243)	Gelatin	None (glaucoma drainage)	Subconjunctival implant	Glaucoma
Cypass (243)	Polyamide	None (glaucoma drainage)	Suprachoroidal implant	Glaucoma

In the intravitreal space, progress has been much slower, with only seven intravitreal polymer systems obtaining regulatory approval for use with a small set of diseases (46, 200, 213, 244). These seven, the Iluvien®, Ozurdex®, Retisert®, Vitrasert®, Yutiq®, Dextenza, and DEXYCU® implants, use a variety of polymers in their construction. Iluvien® and Yutiq® use polyimide implants to deliver fluocinolone acetonide (46, 235). Ozurdex® uses a PLGA matrix that degrades to release dexamethasone (236). Retisert® contains a fluocinolone acetonide tablet in a silicone/PVA elastomer (46, 237). Vitrasert® releases ganciclovir from a PVA/ethylene vinyl acetate system (200). Dextenza suspends dexamethasone in a PEG-fluorescein hydrogel (238). Finally, DEXYCU® makes use of acetyl triethyl citrate gel to deliver suspended dexamethasone (46). Four of these seven are non-degradable implants; Ozurdex®, Dextenza, and DEXYCU® are capable of resorption into the tissue of the eye. This helps to control drug release rate by providing a constant polymer membrane through which drug diffuses into the intravitreal space. However, it also presents challenge of implant removal and replacement once its therapeutic payload is expended, requires surgery and may incur additional health risks for the patient. A search of the Drugs@FDA database indicates that Iluvien, Ozurdex, Yutiq, DEXYCU, Dextenza, and Retisert remain available by prescription, while Vitrasert has been discontinued in the US. There are many more polymer implants in various phases of clinical and laboratory research making use of materials such as PLGA and PEG, indicating that there is significant progress yet to be made in clinical deployment of polymer systems in the vitreal space (20, 200, 226). In addition to recently approved systems such as the Genentech Port Delivery system, Kodiak is currently in phase 3 trials using an injectable biopolymer-antibody conjugate for the treatment of wet AMD and DME, while Aerie is testing biodegradable polymer implants for DME in a phase 2 trial (245, 246). With ongoing efforts in the development of intravitreal microparticles, nanoparticles, and injectable hydrogels, it is likely that intravitreal drug delivery options available to patients and clinicians will become significantly more diverse in the coming years (20, 142, 227, 247).

Subconjunctival drug delivery is a route that has only recently begun to be explored. Despite this, there has been progress in the development of subconjunctival polymer drug delivery systems, with the Ologen® and Xen Gel systems using collagen to construct implants and research efforts into other polymers such as PLGA showing promising results for implant performance (197, 201). However, these implants may pose challenges with discomfort and potential complications, leaving significant room for improvements in the future (197, 199). Research into other polymer systems for subconjunctival delivery is an emerging area, with several research efforts investigating alternative implant polymer compositions, nanoparticle-based delivery systems, and injectable hydrogels for use as drug reservoirs in the subconjunctival space (197, 199, 209, 248–250). However, many of these are still in the early phases of development, and are likely to require further research showing safety and biocompatibility, as well as well-developed animal studies to show efficacy, before they can be put into clinical trials (197).

Finally, while the suprachoroidal route is the least explored for ocular drug delivery, products such as the XIPERE™ system's injectable triamcinolone acetonide solution are nearing market approval (201, 251). In addition to these promising developments in suprachoroidal injections, there are several choroidal devices that have found success in clinical uses. In particular, choroidal shunts made of polymers for the reduction of IOP in glaucoma patients have been the subject of significant investigation as an alternative to subconjunctival drainage, and choroidal port delivery systems have been successful in clinical trials evaluating their efficacy for drug delivery in retinal diseases (19, 252, 253). The ability to build on these innovations and incorporate polymers used in other ocular drug delivery systems will provide a valuable and viable path forward for the development of polymer systems for suprachoroidal injection.

Part of the reason that only a small number of synthetic polymers are being used in ocular drug delivery applications is regulatory hurdles. Even using FDA-approved therapeutics, these drug-device combinations must perform more testing than traditional medical devices through a 510(k) approval pathway with the FDA. Other challenges include the fact that the polymer delivery system likely changes the required therapeutic dose, generally leading to less therapeutic need due to reduced therapeutic waste. For example, when polymer delivery systems are employed, drug retention on the cornea improves significantly compared to non-polymer delivery systems (158). The reduction in necessary dose is not usually known until preclinical or clinical studies are conducted. Dosing at lower levels can be estimated using effective therapeutic concentrations, but long-term stability and therapeutic shelf-life are still concerns that must be addressed prior to approval.

## Future Directions

While polymers have been used in ocular drug delivery for decades, with the first polymer intravitreal implants receiving approval in 1996 and topical applications making use of them since the 1970s, many applications of polymers in ocular drug delivery systems are still in the early stage of development, with significant untapped innovation that could lead to drastic improvements in the capability, quality, and ease of these treatments (213). The next decade will see a large increase in preclinical and clinical trials of polymer-based ocular drug delivery systems. Eyedrop systems have found some success in the development and clinical approval of polymers designed to extend the residence time of the drop on the corneal surface (207). However, continued challenges in corneal penetration leave room for further exploration. Ongoing research into the translation of technologies such as nanomicelles and gelling agents to clinical applications seeks to further improve the efficacy of eyedrops as a delivery system (110, 254). Topical delivery to treat posterior segment diseases is also an area worth exploring to benefit patients. Intravitreal injections and implants have begun to embrace polymers as a method of increasing delivery duration with the development of polymer implants. Intravitreal implants, however, can be difficult to properly position and more difficult to extract once depleted. Further developments in biodegradable implants like Ozurdex®, as well as the development of alternative systems such as *in-situ* forming hydrogels, are likely to create less invasive intravitreal systems with similar capability to improve efficiency and reduce injection frequency. Subconjunctival and suprachoroidal injections and implants, as the youngest types of delivery systems, benefit from developments in other fields and are well-positioned to develop quickly once research locates optimal polymer formulations for both injectable solutions and implantable systems. For all of these methods, obtaining regulatory approval will be perhaps their most significant challenge. Many ocular drug delivery systems are listed in the FDA's drug databases, indicating that they were required to pass the FDA's drug approval process rather than obtaining device certification before reaching the open market. Despite this challenge in obtaining approval, dozens of polymer drug delivery systems are currently in clinical or preclinical trials for ocular applications, highlighting the immense potential many see for future growth in this field (20, 146, 255). Overall, the future is bright for the use of polymers in ocular drug delivery systems, with a solid foundation of clinical technologies, dozens of registered clinical trials evaluating next-generation delivery systems for even higher efficiency, and further investigative research developing applications of new polymer science in ocular delivery.

## Author Contributions

MA and KS-R were responsible for study conception. MA, RL, EH, and KS-R: literature review, analysis, interpretation of results, and writing were conducted. MA was primarily responsible for drafting the manuscript. All authors reviewed the results and approved the final version of the manuscript.

## Funding

We would like to acknowledge the Ohio State University College of Engineering, the Ohio Lions Eye Research Foundation, and the Research to Prevent Blindness Young Investigator Student Fellowship Award for Female Scholars in Vision Research for funding.

## Conflict of Interest

KS-R consults for and has equity interest in Vitranu, Inc., who has licensed ocular drug delivery technologies from her lab. KS-R has patent applications for ocular drug delivery technologies. The remaining authors declare that the research was conducted in the absence of any commercial or financial relationships that could be construed as a potential conflict of interest.

## Publisher's Note

All claims expressed in this article are solely those of the authors and do not necessarily represent those of their affiliated organizations, or those of the publisher, the editors and the reviewers. Any product that may be evaluated in this article, or claim that may be made by its manufacturer, is not guaranteed or endorsed by the publisher.
